# Clindamycin-Induced Dermatitis and the Sparing Phenomenon: A Case Report

**DOI:** 10.7759/cureus.91314

**Published:** 2025-08-30

**Authors:** Noah Gordon, Michelle Zydeck, Ruba Odeh

**Affiliations:** 1 Internal Medicine, Advocate Lutheran General Hospital, Park Ridge, USA; 2 Infectious Disease, Advocate Lutheran General Hospital, Park Ridge, USA

**Keywords:** adverse drug events, clindamycin, dermatitis medicamentosa, drug-induced dermatitis, drug-induced rash, drug rash, drug reaction with eosinophilia and systemic symptoms (dress) syndrome, koebner phenomenon, sparing phenomenon

## Abstract

Drug-induced dermatitis is a type IV, T-cell-mediated, hypersensitivity reaction characterized by the development of a rash in response to drug exposure. The sparing phenomenon, where drug-induced rash spares areas of recent skin disease, is a recognized but relatively rare clinical observation. In this report, a 62-year-old woman presented to the emergency department with drug-induced dermatitis secondary to clindamycin that spared a recent site of *Pasteurella multocida* cellulitis. This case visually highlights the complex interplay between T-cell activation and the local immune environment.

## Introduction

Adverse cutaneous drug reactions are frequently seen in clinical practice, affecting up to 10% of hospitalized patients [1]. Most adverse drug rashes are mild. However, up to 2% may progress to severe cutaneous adverse reactions (SCARs), which are life-threatening [1,2]. SCARs include drug reaction with eosinophilia and systemic symptoms (DRESS) syndrome, acute generalized exanthematous pustulosis (AGEP), Stevens-Johnson syndrome (SJS), and toxic epidermal necrolysis (TEN).

The most common presentation of a cutaneous drug reaction is a morbilliform symmetric rash with confluent erythematous macules and papules that spare the palms and soles, generally referred to as drug-induced dermatitis. Drug-induced dermatitis represents a delayed type IV hypersensitivity reaction, with severity ranging from mild to extensive eruptions. Management typically involves discontinuing the causative agent and providing supportive care.

Clindamycin is a well-recognized cause of cutaneous adverse reactions. In a large study of 3,896 clindamycin administrations at a single US hospital, the incidence of such reactions was 0.47%, with most presenting as delayed cutaneous eruptions [3].

Beyond the typical patterns of drug-induced dermatitis, clinicians may also encounter unusual cutaneous phenomena that deviate from expected distributions. One such observation is the sparing phenomenon, in which previously affected or distinct areas of skin remain uninvolved during new dermatologic lesions or disease. The prevalence of this finding is unknown but has been described in isolated cases in medical literature. Documented examples include patients with alopecia areata sparing areas with psoriasis, dermatophytosis sparing congenital nevi, and leukocytoclastic vasculitis sparing tattoos [4-6]. We report the case of a 62-year-old woman who developed clindamycin-induced dermatitis that spared a recent site of cellulitis.

## Case presentation

A 62-year-old woman with a medical history of multiple sclerosis on dimethyl fumarate presented to the emergency department (ED) two days after being scratched by her pet cat on the anterior region of the distal left lower extremity. In the days following the scratch, her leg became increasingly swollen and red, leading to her decision to seek medical care. In the ED, she remained clinically stable without concern for septic shock. Therefore, the decision was made to proceed with oral antibiotics and transitioning care to the outpatient setting. Due to her penicillin allergy, she was prescribed levofloxacin and clindamycin as empiric treatment. The following day, she was notified that her blood culture tested positive for *Pasteurella multocida* and she would require hospitalization for appropriate medical treatment and monitoring (Table 1).

**Table 1:** Blood culture and susceptibility pattern on initial presentation for cellulitis. Growth detected from aerobic bottle after 15 hours.

Blood culture	Patient value	Reference range
Culture	Pasteurella multocida	No growth
Gram smear	Gram-negative Bacillus	No growth
Antibiotic susceptibility	Interpretation	Value (µg/mL)
Ampicillin	Susceptible	0.25
Amoxicillin/clavulanic acid	Susceptible	0.5
Ceftriaxone	Susceptible	<0.016
Levofloxacin	Susceptible	0.012
Penicillin G	Susceptible	0.19
Trimethoprim/sulfamethoxazole	Susceptible	0.5

**Figure 1:**
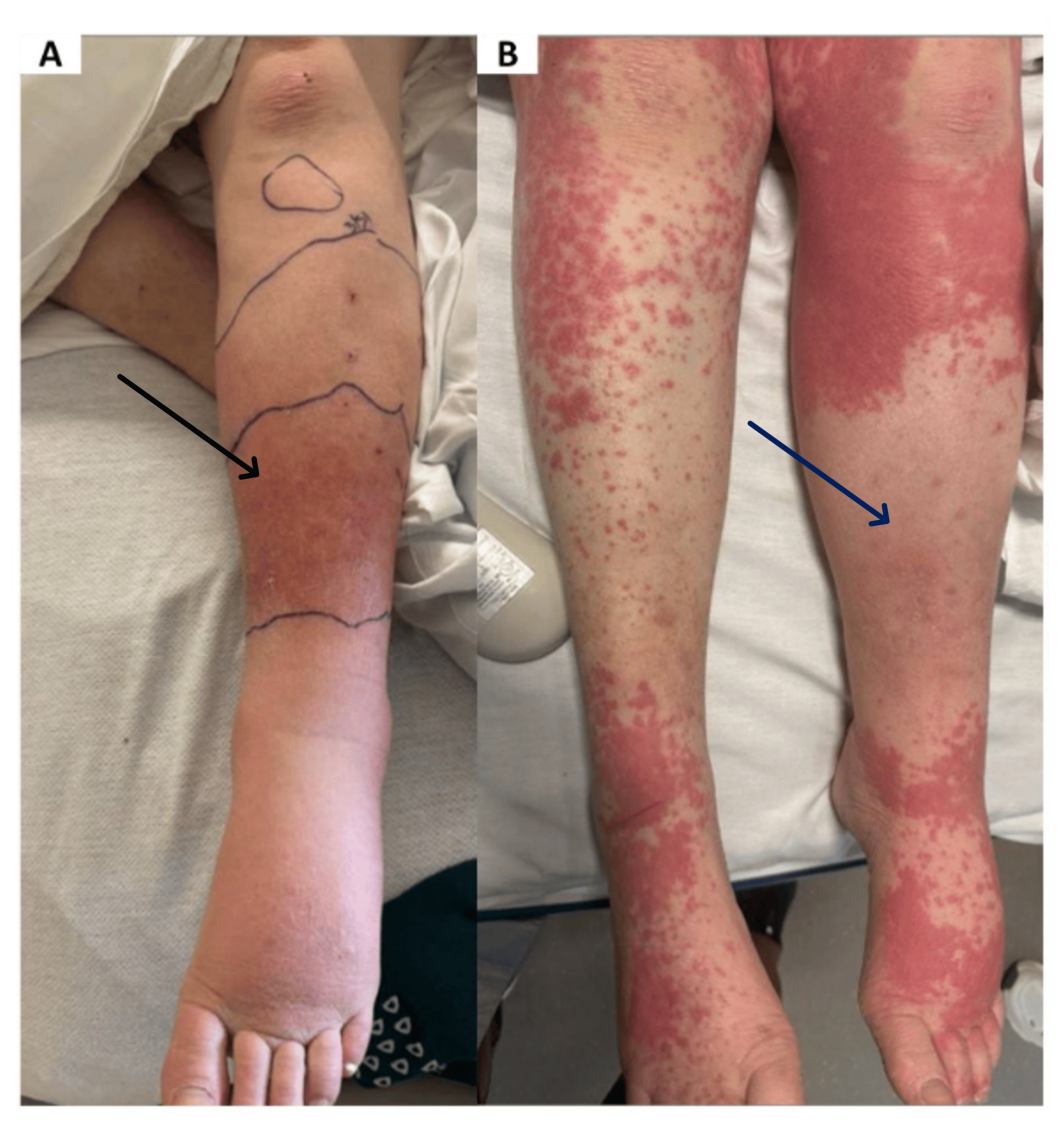
Left lower extremity comparing the distribution of cellulitis versus drug-induced dermatitis. (A) Pasteurella multocida cellulitis (black arrow) outlined with marker on day 2 of intravenous ceftriaxone. (B) Drug-induced dermatitis with maculopapular rash sparing the area of cellulitis (blue arrow).

On evaluation, she was hemodynamically stable and afebrile. The physical exam was significant for a linear scratch mark approximately 1 cm on visual estimation over the left anterior leg surrounded by warm, erythematous skin ascending to the knee (Figure 1). Over the course of four days on ceftriaxone, she remained hemodynamically stable with negative surveillance cultures and improving cellulitis. Therefore, she was discharged with levofloxacin to complete the full course of antibiotic therapy.

Five days following discharge, she returned to the ED with complete resolution of her cellulitis, although there was a new diffuse, coalescing maculopapular rash. The new rash started about one day following discharge when she resumed oral antibiotics. It first appeared on her bilateral upper extremities and chest, spreading downwards rapidly with associated pruritus. There were no blisters or bullae present. Notably, the rash spared the oral mucosa, palms, and soles and her previous area of cellulitis and was without sloughing (Figure 1). On clarification, with the assistance of clinical pharmacy, we discovered she had been accidentally taking the previously prescribed clindamycin, not levofloxacin as instructed on discharge. Laboratory evidence was significant for acute kidney injury (Table 2) and absolute eosinophilia (Table 3).

**Table 2:** Complete metabolic panel comparing values of patient presentation for lower extremity cellulitis compared to subsequent encounter for drug-induced dermatitis.

Complete metabolic panel	Patient value (cellulitis)	Patient value (rash)	Reference range
Sodium (mmol/L)	144	135	135-145
Potassium (mmol/L)	3.7	4.4	3.4-5.1
Chloride (mmol/L)	107	100	97-110
Carbon dioxide (mmol/L)	27	22	21-32
Anion gap (mmol/L)	14	17	7-19
Glucose (mg/dL)	136	135	77-99
Blood urea nitrogen (mg/dL)	20	30	6-20
Creatinine (mg/dL)	0.60	1.12	0.51-0.95
Glomerular filtration rate	>90	56	≥60
Calcium (mg/dL)	8.9	9.1	8.4-10.2
Bilirubin, total (mg/dL)	1.1	0.5	0.2-1.0
Aspartate aminotransferase (Units/L)	26	37	≤37
Alanine aminotransferase (Units/L)	29	47	<64
Alkaline phosphatase (Units/L)	84	108	45-117
Albumin (g/dL)	3.8	2.6	3.4-5.0
Protein, total (g/dL)	7.4	7.1	6.4-8.2

**Table 3:** Complete blood count comparing values of patient presentation for lower extremity cellulitis compared to subsequent encounter for drug-induced dermatitis. Notably, elevation in white blood cells in part due to outpatient steroid prescription administered prior to subsequent emergency department encounter.

Complete blood count	Patient value (cellulitis)	Patient value (rash)	Reference range
White blood cells (K/mcL)	7.5	35.9	4.2-11.0
Red blood cells (mil/mcL)	5.16	5.07	4.00-5.20
Hemoglobin (g/dL)	13.3	13.4	12.0-15.5
Hematocrit (%)	43.5	41.6	36.0-46.5
Mean corpuscular volume (fl)	84.3	82.1	78.0-100.0
Platelets (K/mcL)	216	546	140-450
Neutrophil (%)	84	92	-
Lymphocytes (%)	9	3	-
Monocytes (%)	6	2	-
Eosinophils (%)	1	2	-
Basophils (%)	0	0	-
Immature granulocytes (%)	0	1	-
Absolute neutrophils (K/mcL)	6.3	32.9	1.8-7.7
Absolute lymphocytes (K/mcL)	0.6	1.2	1.0-4.0
Absolute monocytes (K/mcL)	0.5	0.6	0.3-0.9
Absolute eosinophils (K/mcL)	0.1	0.9	0.0-0.5
Absolute basophils (K/mcL)	0.0	0.1	0.0-0.3
Absolute immature granulocytes (K/mcL)	0.0	0.3	0.0-0.2

Despite these laboratory abnormalities, her constellation of symptoms was not severe enough to qualify as a DRESS syndrome. Treatment consisted of a prednisone taper, and clindamycin administration was promptly stopped. Over the course of three days, the rash gradually faded, and pruritus completely resolved.

## Discussion

In this patient, clindamycin likely acted as a hapten, covalently binding to host proteins to form novel drug-protein complexes. These drug-protein conjugates, previously unrecognized by the immune system, primed antigen-specific memory T-cells [7]. Upon re-exposure, this immunologic memory triggered a rapid, coordinated T-cell-mediated response, manifesting clinically as a diffuse, pruritic rash consistent with drug-induced dermatitis. 

Interestingly, despite the widespread immune activation, this case also revealed a striking immunologic feature: the sparing phenomenon. This case report demonstrates the sparing phenomenon, an immunologically induced T-cell refractory period that we observed in a 62-year-old woman with *Pasteurella multocida* cellulitis, illustrated by the localized absence of drug-induced dermatitis. This refractory period reflects a protective immune mechanism aimed at promoting tissue healing. As immunologic insults resolve, the immune system employs regulatory T-cells (Tregs) to downregulate inflammation and prevent further immune activation. This is accomplished through multiple mechanisms: the secretion of anti-inflammatory cytokines such as interleukin (IL)-10 and transforming growth factor (TGF)-β, the expression of cytotoxic T-lymphocyte-associated antigen 4 (CTLA-4) to modulate dendritic cell activity, and the release of extracellular vesicles carrying microRNA that enhance IL-10 production and suppress IL-6 production in dendritic cells [8-11]. 

Clinically, the sparing phenomenon may serve as a subtle but meaningful diagnostic clue, indicating localized immune downregulation. Although in this case there were no obvious benefits or harms resulting from this altered immune environment, this phenomenon is not entirely benign. The downregulation of the immune response creates a microenvironment that may be less capable of defending against foreign pathogens [12]. Thus, while this response aims to prevent excessive immune activation, its unintended consequence can be fertile ground for infectious agents to propagate unchallenged. While we visualized this immune downregulation in the skin of the leg, the phenomenon is not exclusive to the integument. This pathophysiology also plays a central role in the classically described "double sickening," in which, during the recovery phase of a viral pneumonia, such as influenza, secondary bacterial infections superimpose and develop into a new pneumonia [13,14]. 

In summary, this case of clindamycin-induced dermatitis illustrates a classic T-cell-mediated hypersensitivity reaction initiated by hapten formation. Concurrently, the sparing phenomenon highlights the critical role of regulatory mechanisms, particularly Tregs, in tempering immune responses to prevent tissue damage and facilitate healing. This duality between immune activation and regulatory control underscores the immune system's delicate balance between defense and tolerance.

Further research into the mechanisms governing immune sparing and its clinical sequelae may pave the way for novel approaches to managing drug reactions and post-infectious immune modulation. A deeper understanding of Treg function in this context may also inform strategies to enhance immune resolution without compromising host defense, particularly in settings where tissue repair and pathogen control must coexist. 

## Conclusions

This case underscores the complexity of immune response and regulation. Specifically, the presentation of clindamycin-induced dermatitis, coupled with a distinct sparing phenomenon, highlights the dynamic interplay between immune activation and localized immunosuppression. Recognition of such patterns not only aids diagnostic accuracy but also provides insight into underlying immunologic processes, particularly the role of regulatory T-cells in promoting tissue healing despite potentially compromising local immune defense. As our understanding of these mechanisms evolves, it may inform future therapeutic strategies aimed at balancing immune resolution with infection control in both dermatologic and systemic contexts. Moreover, this case also emphasizes the importance of detailed medication reconciliation and follow-up.
